# Association of accelerometer-derived sleep measures with lifetime psychiatric diagnoses: A cross-sectional study of 89,205 participants from the UK Biobank

**DOI:** 10.1371/journal.pmed.1003782

**Published:** 2021-10-12

**Authors:** Michael Wainberg, Samuel E. Jones, Lindsay Melhuish Beaupre, Sean L. Hill, Daniel Felsky, Manuel A. Rivas, Andrew S. P. Lim, Hanna M. Ollila, Shreejoy J. Tripathy

**Affiliations:** 1 Krembil Centre for Neuroinformatics, Centre for Addiction and Mental Health, Toronto, Canada; 2 Institute for Molecular Medicine Finland (FIMM), University of Helsinki, Helsinki, Finland; 3 University of Exeter Medical School, Exeter, United Kingdom; 4 Campbell Family Mental Health Research Institute, Centre for Addiction and Mental Health, Toronto, Canada; 5 Institute of Medical Sciences, University of Toronto, Toronto, Canada; 6 Department of Psychiatry, University of Toronto, Toronto, Canada; 7 Department of Physiology, University of Toronto, Toronto, Canada; 8 Biostatistics Division, Dalla Lana School of Public Health, University of Toronto, Toronto, Canada; 9 Department of Genetics, Stanford University, Stanford, California, United States of America; 10 Sunnybrook Health Sciences Centre, Toronto, Canada; 11 Division of Neurology, Department of Medicine, University of Toronto, Toronto, Canada; 12 Center for Genomic Medicine, Massachusetts General Hospital, Boston, Massachusetts, United States of America; 13 Program in Medical and Population Genetics, Broad Institute, Cambridge, Massachusetts, United States of America; 14 Department of Anesthesia, Critical Care and Pain Medicine, Massachusetts General Hospital and Harvard Medical School, Boston, Massachusetts, USA; Harvard Medical School, UNITED STATES

## Abstract

**Background:**

Sleep problems are both symptoms of and modifiable risk factors for many psychiatric disorders. Wrist-worn accelerometers enable objective measurement of sleep at scale. Here, we aimed to examine the association of accelerometer-derived sleep measures with psychiatric diagnoses and polygenic risk scores in a large community-based cohort.

**Methods and findings:**

In this post hoc cross-sectional analysis of the UK Biobank cohort, 10 interpretable sleep measures—bedtime, wake-up time, sleep duration, wake after sleep onset, sleep efficiency, number of awakenings, duration of longest sleep bout, number of naps, and variability in bedtime and sleep duration—were derived from 7-day accelerometry recordings across 89,205 participants (aged 43 to 79, 56% female, 97% self-reported white) taken between 2013 and 2015. These measures were examined for association with lifetime inpatient diagnoses of major depressive disorder, anxiety disorders, bipolar disorder/mania, and schizophrenia spectrum disorders from any time before the date of accelerometry, as well as polygenic risk scores for major depression, bipolar disorder, and schizophrenia. Covariates consisted of age and season at the time of the accelerometry recording, sex, Townsend deprivation index (an indicator of socioeconomic status), and the top 10 genotype principal components. We found that sleep pattern differences were ubiquitous across diagnoses: each diagnosis was associated with a median of 8.5 of the 10 accelerometer-derived sleep measures, with measures of sleep quality (for instance, sleep efficiency) generally more affected than mere sleep duration. Effect sizes were generally small: for instance, the largest magnitude effect size across the 4 diagnoses was β = −0.11 (95% confidence interval −0.13 to −0.10, *p* = 3 × 10^−56^, FDR = 6 × 10^−55^) for the association between lifetime inpatient major depressive disorder diagnosis and sleep efficiency. Associations largely replicated across ancestries and sexes, and accelerometry-derived measures were concordant with self-reported sleep properties. Limitations include the use of accelerometer-based sleep measurement and the time lag between psychiatric diagnoses and accelerometry.

**Conclusions:**

In this study, we observed that sleep pattern differences are a transdiagnostic feature of individuals with lifetime mental illness, suggesting that they should be considered regardless of diagnosis. Accelerometry provides a scalable way to objectively measure sleep properties in psychiatric clinical research and practice, even across tens of thousands of individuals.

## Abstract 2

In a cross-sectional study, Michael Wainberg and colleagues investigate the association between accelerometer-derived sleep measures and lifetime psychiatric diagnoses.

## Author summary

**Why was this study done?:**

- Sleep problems are both symptoms of and risk factors for many mental health conditions.
- This study aimed to determine how objectively measured sleep differs among individuals with lifetime psychiatric diagnoses.

**What did the researchers do and find?:**

- This cohort study of 89,205 individuals from the UK Biobank analyzed 10 accelerometer-derived sleep measures.
- The study found a rich suite of associations with lifetime diagnoses of psychopathology and psychiatric polygenic risk scores, though effect sizes were generally small.

**What do these findings mean?:**

- Sleep pattern differences are the norm among patients with lifetime psychiatric illness.
- Accelerometry provides a scalable way to objectively measure such differences in psychiatric research and practice.
- Limitations include the use of accelerometer-based sleep measurement and the time lag between psychiatric diagnoses and accelerometry.

## Introduction

Sleep is fundamental to mental health. Poor sleep is not just a hallmark of psychiatric disorders, but can be a causal risk factor as well [1]. Sleep interventions can lessen depression [2] and posttraumatic stress disorder [3] symptoms, prevent psychotic experiences [4,5], and improve psychological well-being and quality of life [6].

In psychiatry, sleep properties are often ascertained via self-report: for instance, self-reported sleep quality is a component of nearly every depression rating scale, including the HAM-D [7] and Montgomery–Asberg [8]. However, self-reported measures of sleep do not always correlate well with direct physiological measurements: prior work has found that a typical person may overestimate [9,10] or underestimate [11,12] their sleep duration by up to 75 minutes, relative to direct measurement. This divergence may be especially large among psychiatric patients: individuals with depression are less accurate at reporting sleep quality and duration than healthy controls [13]. Thus, when studying sleep in a psychiatric context, objective measurement may be a useful complement to self-report. While lab-based polysomnography remains the gold standard for sleep measurement, it is ill-suited to long-term or home use, and spending a night in a sleep clinic with multiple electrodes attached to one’s body may not be conducive to a good night’s sleep. Wrist-based accelerometry (also called actigraphy) is a reasonably accurate and much more versatile and scalable alternative [14–19].

Historically, most accelerometry studies of sleep and mental illness have relied on highly selected samples of tens to hundreds of individuals [20]. Recently, the UK Biobank collected 7-day accelerometry recordings from over 100,000 participants [21], providing an unprecedented opportunity to study the interplay between sleep and mental health across a broad cross-section of the community. Researchers have used this dataset to determine that circadian dysrhythmia is correlated with mood disorders and subjective well-being [20] and genetically correlated with mood instability [22] and that insomnia, chronotype [23], sleep duration [24], and daytime sleepiness [25] are genetically correlated with lifetime prevalence of several psychiatric disorders.

Yet despite recognition that insomnia and disturbed sleep are transdiagnostic processes [26,27] that cut across conventional diagnostic boundaries, the relationship between objectively measured sleep and mental health has rarely been studied from a transdiagnostic perspective—and even then, often only for a single sleep property at a time and in a small sample. To illustrate this research gap, we searched PubMed for studies of objectively measured sleep in a psychiatric context, using the search terms “sleep AND (polysomnography OR accelerometry OR actigraphy) AND (depression OR anxiety OR bipolar OR schizophrenia),” and identified 2,923 articles meeting these criteria. However, after narrowing our search criteria to studies considering all 4 disorders—“sleep AND (polysomnography OR accelerometry OR actigraphy) AND (depression **AND** anxiety **AND** bipolar **AND** schizophrenia)”—we identified only 4 articles: 2 reviews [28,29], a case series of 58 patients [30], and a cohort study of 110 patients also focused on sleep apnea [31].

Here, we address this research gap by performing an “all-by-all” analysis of sleep and mental health across 89,205 UK Biobank participants. Specifically, we investigate the associations of 10 sleep measures—including bedtime and wake-up time, sleep duration, number of awakenings, and variability in bedtime and sleep duration—with 4 lifetime psychiatric diagnoses—major depressive disorder, anxiety disorders, bipolar disorder/mania, and schizophrenia spectrum disorders—as well as polygenic risk scores for major depression, bipolar disorder, and schizophrenia.

## Methods

This study is reported as per the Strengthening the Reporting of Observational Studies in Epidemiology (STROBE) guideline (**S1 Checklist**). The study did not have a prospective protocol or analysis plan.

### Cohort

Accelerometry recordings were gathered from 103,688 participants in the UK Biobank, a community-based prospective cohort study, between 2013 and 2015 [21]. Briefly, participants were provided with an Axivity AX3 triaxial accelerometer by mail and asked to wear it on their dominant wrist for 7 days, starting immediately after receiving it in the mail. These data have been made available as Data-Field 90001 of the UK Biobank (“Acceleration data—cwa format”).

Of these 103,688, participants were excluded if they did not wear the accelerometer for every one of the 24 hours in a day on at least one of the days (Data-Field 90084, “Unique hours of wear in a 24 hour cycle (scattered over multiple days)”; *N =* 4,345); if their accelerometer was not well calibrated (Data-Field 90016, “Data quality, good calibration”; *N =* 11); if their wear period included a DST change (Data-Field 90018, “Daylight savings crossover”; *N* = 4,543); if they woke up in the afternoon on an average day (for instance, shift workers; *N* = 137); or if fewer than 2 days during the 7-day wear period were valid (see below; *N* = 6,020). Due to the inclusion of analyses involving polygenic risk scores, participants were also excluded if they had greater than 2% genotype missingness (Data-Field 22005, “Missingness”), a mismatch between genetic sex and self-reported sex, sex chromosome aneuploidy, or were flagged as “Outliers for heterozygosity or missing rate” (Data-Field 22027). Self-reported white participants (according to Data-Field 21000, “Ethnic background”; *N =* 86,513) were used for the primary analysis, with replication in a much smaller number of self-reported non-white participants (*N* = 2,692), for a total of 89,205 participants. Replication was also performed stratified by sex, among self-reported white females (*N* = 48,562) and males (*N* = 37,951).

### Accelerometry data processing

Accelerometry recordings were temporally segmented into sleep and activity bouts using an accelerometry software toolkit (github.com/activityMonitoring/biobankAccelerometerAnalysis) specifically designed for the UK Biobank [32,33]. As described previously, this segmentation was performed by a machine learning classifier consisting of a random forest, the predictions of which are temporally smoothed by a hidden Markov model. This classifier was trained on an external, labeled dataset of accelerometer recordings. For our analyses, we ignored distinctions between activities and classified each bout as either “sleep” or “wake.” Bouts for times when the accelerometer was not worn were probabilistically imputed; we labeled these bouts as “sleep” if the imputed probability of sleep was greater than 0.5, and “wake” otherwise.

While this segmentation is sufficient to determine the start and end time of each sleep and wake bout, it does not annotate which bouts make up the primary sleep period (usually at night) and which are just naps. To do this, we used steps 7 to 10 of the Heuristic algorithm looking at Distribution of Change in Z-Angle (HDCZA) algorithm implemented in the widely used GGIR accelerometry toolkit [34]: following GGIR, we defined each day’s primary sleep period as the longest time period containing sleep bouts of at least 30 minutes separated by gaps of no more than 60 minutes. (While this definition is commonly used in the field, there is no single correct definition of what should constitute sleep inside versus outside the primary sleep period, particularly for individuals with highly fragmented sleep.) A “day” was defined as the period from 3 PM to the following 3 PM. Days were deemed invalid and discarded if their primary sleep period crossed one of the 3 PM day boundaries, if all the day’s sleep periods were less than 30 minutes, or if more than 10% of the day’s data was imputed.

Having defined each day’s primary sleep period, we defined 10 sleep measures based on the timings and lengths of the sleep and wake bouts inside and outside of this period (**Table 1**). These measures are similar to those used in previous accelerometry and polysomnography studies [35,36]. All measures were quantified as medians (or median absolute deviations, for the variability measures) across days, to be robust to outliers. To keep the focus on sleep, we do not include activity features, nor the L5 and M10 measures of circadian rhythmicity used in a previous study of the UK Biobank [20], which are based on both sleep and activity.

**Table 1:** The 10 sleep features and their definitions. Medians and mean absolute deviations are taken across all valid days.

Sleep feature	Definition
**Bedtime**	Median start time of primary sleep period, expressed in hours since midnight of the previous day.
**Wake-up time**	Median end time of primary sleep period, expressed in hours since midnight of the previous day.
**Sleep duration**	Median total duration of sleep bouts during the primary sleep period.
**WASO**	Median total duration of wake bouts during the primary sleep period.
**Sleep efficiency**	Median fraction of the primary sleep period spent asleep, i.e., 1—WASO / (wake-up time—bedtime).
**Number of awakenings**	Median number of wake bouts during the primary sleep period.
**Duration of longest sleep bout**	Median length of the longest sleep bout during the primary sleep period.
**Number of naps**	Median number of >30-minute sleep periods outside the primary sleep period.
**Variability in bedtime**	Median absolute deviation of bedtime across all valid days.
**Variability in sleep duration**	Median absolute deviation of sleep duration across all valid days.

WASO, wake after sleep onset.

### Inpatient psychiatric diagnoses

These 10 sleep measures were tested for association with 4 lifetime inpatient psychiatric diagnoses from any time before the date of accelerometry: schizophrenia spectrum disorders (International Classification of Diseases [ICD] codes F20-F29), bipolar disorder/mania (F30, F31), major depressive disorder (F32, F33), and anxiety disorders (F40, F41). Inpatient diagnoses and their dates were derived from the “hesin_diag” table of the inpatient records provided by the UK Biobank (Data-Field #41234, “Records in HES inpatient diagnoses dataset”).

To mitigate contamination of the control group, we excluded participants with preexisting primary care diagnoses (available for approximately 45% of the cohort), death record-based diagnoses, and/or self-reported clinician diagnoses of the same disorder, according to the “Source of report of [ICD code]” fields provided with the UK Biobank, for instance, Data-Field 130895, “Source of report of F32 (depressive episode).” We also excluded participants whose first inpatient diagnosis of the disorder was after the date of accelerometry. For instance, when computing associations with inpatient major depressive disorder, we excluded participants with primary care, death record-based, or self-reported major depressive disorder diagnoses, or whose first inpatient diagnosis of major depressive disorder was after the date of accelerometry.

### Polygenic risk scores

The 10 sleep measures were also associated with polygenic risk scores derived from public genome-wide association study results for major depression [37], bipolar disorder [38,39], and schizophrenia [40] across self-reported white participants. The UK Biobank’s imputed genotypes were filtered using version 2.0 of the *plink* software [41]. Nonautosomal variants, duplicates, indels, and variants with imputation INFO score less than 0.8 were removed, as were variants with Hardy–Weinberg equilibrium *p*-value less than 10^−10^, over 5% missingness, minor allele frequency below 0.1% across self-reported white participants.

The polygenic risk scores were then calculated. Summary statistics were first harmonized with the UK Biobank imputed genotypes with respect to reference/alternate allele and strand, using the allele harmonization framework from munge_sumstats.py in the *ldsc* software package [42]. Ambiguous variants (A/T, C/G, G/C, T/A) and variants missing from UK Biobank were excluded. Summary statistics were then subset to *p* < 0.05, a threshold found to be most predictive across self-reported white participants in the UK Biobank (**S1 Fig**). Frequency-informed linkage disequilibrium (LD) pruning to r^2^ > 0.2 across the self-reported white participants was then performed using a 500-kb sliding window. The remaining variants constituted the trait’s polygenic risk score, with the variants’ effect sizes (β coefficients for educational attainment, log odds ratios for the other 3 case–control studies) constituting the weights of the score. Finally, polygenic risk scores were scored on each individual in the study cohort by summing, across the variants in the polygenic risk score, the variant’s weight times the individual’s number of effect alleles of that variant; missing genotypes were mean imputed.

### Association analyses

Association tests were performed by linearly regressing the outcome variable (sleep measures) on the exposure variable (psychiatric diagnoses or polygenic risk scores). Covariates consisted of age and season at the time of the accelerometry recording, sex, Townsend deprivation index (an indicator of socioeconomic status), and the top 10 genotype principal components. Benjamini–Hochberg correction [43] was performed at a false discovery rate (FDR) threshold of 5%.

### Analyses of self-reported sleep properties

As a secondary analysis, we considered 6 self-reported sleep properties (**S1 Table**) ascertained at baseline assessment between 2006 and 2010, approximately a half decade earlier than the accelerometry. We first assessed the concordance between self-reported sleep properties and accelerometry-derived sleep measures, by linearly regressing each accelerometry-derived measure (as the dependent variable) on each self-reported sleep property (as the independent variable) across all 77,232 self-reported white participants with both types of sleep properties, using the same covariates as above.

Next, we performed the same battery of associations with psychiatric diagnoses and polygenic risk scores, with the following differences from the primary analysis. First, we analyzed all 400,771 self-reported white participants with self-reported sleep properties and genotype data, not just the 89,205 with accelerometry. Second, we excluded participants with inpatient diagnoses after the baseline assessment, rather than after the date of accelerometry. Third, instead of including the age and season of accelerometry as covariates, we include the age at baseline assessment. Aside from these changes, this secondary analysis was conducted identically to the primary analysis.

### Ethics statement

This study is a reanalysis of the UK Biobank cohort, which obtained ethical approval and informed consent from study participants as described in the flagship UK Biobank publication [44]. This study was conducted under the auspices of UK Biobank application 61530, “Multimodal subtyping of mental illness across the adult lifespan through integration of multiscale whole-person phenotypes.”

## Results

### Accelerometer-derived sleep measures across 89,205 individuals

**Fig 1:**
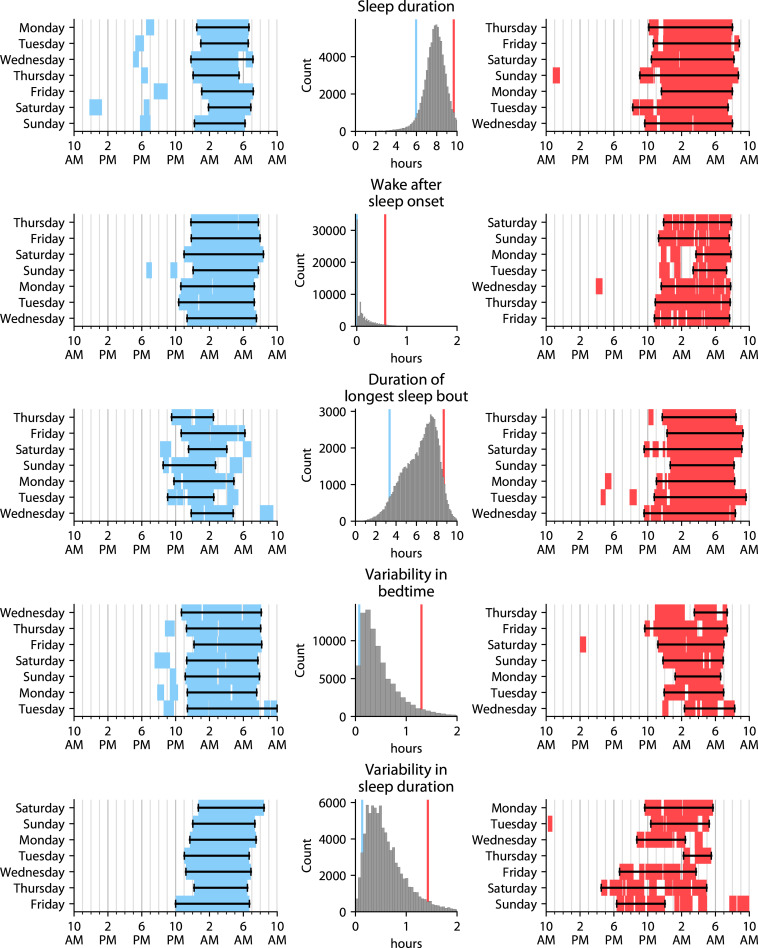
Distributions and exemplar individuals for various sleep measures. Each row’s middle panel shows a 100-bin histogram and Gaussian kernel density estimate of a particular sleep measure across the self-reported white participants. For each measure, 2 exemplar individuals were chosen: one at the 5th percentile (plotted to the left of the histogram), and one at the 95th percentile (plotted to the right of the histogram). The blue (left) and red (right) lines on the histograms denote the 5th and 95th percentiles, i.e., where these 2 exemplar individuals are located on the distribution. In the exemplar plots, blue/red blocks indicate sleep bouts, and black lines with bars indicate each day’s primary sleep period. Days of the week are ordered differently for different exemplars because some people started the accelerometry on different days.

We analyzed accelerometry data from 89,205 participants. Our primary analysis used the largest ancestry group, self-reported white (*N =* 86,513); replication in the much smaller number of self-reported non-white participants (*N* = 2,692) and stratified by sex is discussed in the final subsection of the Results. Characteristics of participants with and without each of the 4 psychiatric diagnoses, for the self-reported white cohort used in the primary analysis, are shown in Table 2. We derived 10 sleep measures from these accelerometry data (**Table 1**, **Fig 1**, **S2 Fig**): bedtime, wake-up time, sleep duration, wake after sleep onset (WASO; the total time spent awake between bedtime and wake-up time), sleep efficiency (the fraction of time spent asleep between bedtime and wake-up time), number of awakenings, duration of longest sleep bout, number of naps, variability in bedtime, and variability in sleep duration.

**Table 2:** Cohort information.

	Major depressive disorder (F32-F33)		Anxiety disorders (F40-F41)		Bipolar disorder/mania (F30-F31)		Schizophrenia spectrum disorders (F20-F29)	
	Cases (*N* = 1,476)	Controls (*N* = 77,840)	Cases (*N* = 810)	Controls (*N* = 80,856)	Cases (*N* = 123)	Controls (*N* = 86,193)	Cases (*N* = 71)	Controls (*N* = 86,341)
Age: 43–49	100	5,269	48	5,531	10	5,924	9	5,929
Age: 50–59	437	21,121	226	22,140	43	23,721	35	23,767
Age: 60–69	671	34,835	359	36,219	50	38,520	21	38,594
Age: 70–79	268	16,615	177	16,966	20	18,028	6	18,051
Sex: female	989	42,784	529	44,756	72	48,367	38	48,471
Sex: male	487	35,056	281	36,100	51	37,826	33	37,870
Townsend: least-deprived quintile	226	15,775	142	16,240	18	17,248	6	17,278
Townsend: second-least deprived quintile	245	15,717	139	16,264	14	17,251	8	17,276
Townsend: middle quintile	278	15,614	139	16,199	23	17,241	11	17,275
Townsend: second-most deprived quintile	294	15,579	180	16,167	20	17,252	13	17,276
Townsend: most deprived quintile	433	15,155	210	15,986	48	17,201	33	17,236
2 valid days	25	1,179	14	1,213	4	1,297	0	1,304
3 valid days	40	1,714	33	1,765	7	1,904	3	1,905
4 valid days	74	2,963	34	3,119	6	3,336	5	3,338
5 valid days	155	6,487	72	6,787	16	7,279	9	7,294
6 valid days	343	17,380	175	18,088	21	19,337	10	19,360
7 valid days	839	48,117	482	49,884	69	53,040	44	53,140
Latest inpatient visit: <6 months ago	187	--	117	--	11	--	2	--
Latest inpatient visit: <1 year ago	341	--	212	--	24	--	6	--
Latest inpatient visit: <2 years ago	592	--	352	--	37	--	17	--
Latest inpatient visit: <5 years ago	1,053	--	613	--	73	--	29	--
Latest inpatient visit: <10 years ago	1,315	--	738	--	101	--	48	--
Latest inpatient visit: <20 years ago	1,476	--	810	--	123	--	71	--

Number of cases and controls for each of the 4 disorders in each bracket of age, sex, Townsend deprivation index (a measure of socioeconomic deprivation), and number of valid days of accelerometry (Methods), as well as the time between the most recent inpatient visit for the disorder and the date of accelerometry (only defined for cases). 7,197 participants were excluded from the major depressive disorder analysis, 4,847 for anxiety disorders, 197 for bipolar disorder/mania, and 101 for schizophrenia spectrum disorders (Methods).

To gain insight into the distributions of these sleep measures, we tabulated percentiles of each measure (**Table 3**) across participants with and without a history of any of 4 common inpatient psychiatric diagnoses from before the date of accelerometry: major depressive disorder, anxiety disorders, bipolar disorder/mania, and schizophrenia spectrum disorders. (Depression, anxiety, schizophrenia, and bipolar disorder are the 4 mental health conditions with the greatest global disease burden according to the Global Burden of Disease Study 2019 [45]). The medians (50th percentiles) of these measures were similar between those with and without psychiatric diagnoses: a marginally later bedtime of 11:29 PM instead of 11:19 PM and wake-up time of 7:41 AM instead of 7:24 AM, an identical 99% sleep efficiency, a single awakening, and so on. Differences were larger at one or both extremes, at least for some measures: for instance, the 99th percentile of bedtime was 2:33 AM for those without psychiatric diagnoses, but 4:47 AM for those with diagnoses, while the 99th percentile of wake time was 9:57 AM for those without diagnoses but 10:52 AM for those with.

**Table 3:** Percentiles of sleep measures across individuals with and without prior psychiatric diagnoses (psych. diagn.).

%ile	Psychiatric diagnoses	Bedtime	Wake-up time	Sleep duration	WASO	Sleep efficiency	# awakenings	Longest sleep bout	# naps	Bedtime variability	Sleep duration variability
**1st**	**No**	8:10 PM	4:38 AM	4:21	0:00	89%	0	2:15	0	0:01	0:03
	**Yes**	8:14 PM	4:31 AM	2:48	0:00	85%	0	1:40	0	0:00	0:04
**10th**	**No**	10:05 PM	6:09 AM	6:33	0:00	95%	0	4:03	0	0:06	0:11
	**Yes**	10:00 PM	6:15 AM	5:50	0:00	93%	0	3:14	0	0:07	0:13
**25th**	**No**	10:44 PM	6:47 AM	7:15	0:00	97%	0	5:15	0	0:12	0:18
	**Yes**	10:46 PM	6:57 AM	7:00	0:00	96%	0	4:27	0	0:14	0:21
**50th**	**No**	11:19 PM	7:24 AM	7:54	0:04	99%	1	6:42	0	0:21	0:30
	**Yes**	11:29 PM	7:41 AM	7:53	0:06	99%	1	6:03	0	0:26	0:36
**75th**	**No**	11:57 PM	8:01 AM	8:32	0:12	100%	1	7:41	1	0:37	0:46
	**Yes**	12:18 AM	8:24 AM	8:44	0:18	100%	2	7:30	1	0:45	0:58
**90th**	**No**	12:37 AM	8:36 AM	9:11	0:24	100%	2	8:19	1	0:59	1:08
	**Yes**	1:22 AM	9:11 AM	9:32	0:33	100%	2	8:24	2	1:15	1:24
**99th**	**No**	2:33 AM	9:57 AM	10:45	0:50	100%	4	9:21	2	2:01	2:02
	**Yes**	4:47 AM	10:52 AM	11:34	0:56	100%	4	9:52	3	2:34	2:24

Percentiles were tabulated across the 86,513 self-reported white participants with accelerometry.

WASO, wake after sleep onset; %ile, percentile.

### Association of accelerometer-derived sleep measures with psychiatric diagnoses

We associated these 10 accelerometry-defined sleep measures with 4 ICD-code-based inpatient psychiatric diagnoses (**Table 4**). Three trends were especially striking.

**Table 4:** Association of accelerometer-derived sleep measures with psychiatric diagnoses and polygenic risk scores.

	Bedtime	Wake-up time	Sleep duration	WASO	Sleep efficiency	# awakenings	Longest sleep bout	# naps	Bedtime variability	Sleep dur. variability
**Psychiatric diagnoses**										
**Any psychiatric diagnosis *N* = 9,888**	**0.07** [0.06, 0.09] (7 × 10^−23^)	**0.10** [0.09, 0.11] (1 × 10^−43^)	**−0.02** [−0.03, 0.00] (0.01)	**0.10** [0.09, 0.12] (8 × 10^−47^)	**−0.12** [−0.14, −0.11] (2 × 10^−64^)	**0.10** [0.09, 0.11] (8 × 10^−44^)	**−0.09** [−0.11, −0.08] (1 × 10^−37^)	**0.11** [0.09, 0.12] (7 × 10^−49^)	**0.08** [0.06, 0.09] (2 × 10^−26^)	**0.10** [0.09, 0.12] (2 × 10^−44^)
**Major depressive disorder (F32-F33)** ***N =* 7,197**	**0.07** [0.05, 0.08] (1 × 10^−21^)	**0.09** [0.08, 0.11] (2 × 10^−38^)	**−0.02** [−0.04, −0.01] (0.003)	**0.09** [0.08, 0.11] (1 × 10^−40^)	**−0.11** [−0.13, −0.10] (3 × 10^−56^)	**0.09** [0.07, 0.10] (1 × 10^−34^)	**−0.08** [−0.10, −0.07] (5 × 10^−31^)	**0.10** [0.09, 0.12] (2 × 10^−48^)	**0.08** [0.06, 0.09] (8 × 10^−27^)	**0.10** [0.09, 0.11] (2 × 10^−44^)
**Anxiety (F40-41)** ***N* = 4,847**	**0.03** [0.01, 0.04] (8 × 10^−5^)	**0.04** [0.03, 0.06] (7 × 10^−10^)	0.00 [−0.01, 0.01] (0.9)	**0.04** [0.03, 0.06] (1 × 10^−9^)	**−0.05** [−0.06, −0.03] (6 × 10^−12^)	**0.04** [0.03, 0.06] (2 × 10^−10^)	**−0.04** [−0.05, −0.02] (2 × 10^−7^)	**0.04** [0.02, 0.05] (4 × 10^−7^)	**0.03** [0.01, 0.04] (0.0003)	**0.04** [0.03, 0.06] (1 × 10^−9^)
**Bipolar/mania (F30-F31)** ***N =* 197**	0.01 [0.00, 0.03] (0.07)	**0.02** [0.01, 0.04] (0.0007)	0.00 [−0.01, 0.01] (0.9)	**0.03** [0.01, 0.04] (0.0002)	**−0.03** [−0.05, −0.02] (2 × 10^−6^)	**0.03** [0.02, 0.05] (1 × 10^−6^)	**−0.03** [−0.04, −0.02] (2 × 10^−5^)	**0.04** [0.03, 0.05] (2 × 10^−9^)	**0.02** [0.01, 0.03] (0.002)	**0.02** [0.01, 0.04] (0.001)
**Schizophrenia spectrum (F20-F29)** ***N =* 101**	−0.01 [−0.02, 0.01] (0.4)	**0.03** [0.01, 0.04] (0.0001)	**0.04** [0.02, 0.05] (1 × 10^−7^)	**0.02** [0.01, 0.04] (0.0007)	**−0.02** [−0.04, 0.01] (0.0004)	**0.02** [0.00, 0.03] (0.01)	0.00 [−0.01, 0.02] (0.7)	**0.05** [0.03, 0.06] (1 × 10^−12^)	**0.02** [0.01, 0.03] (0.003)	**0.03** [0.01, 0.04] (7 × 10^−5^)
**Psychiatric polygenic risk scores**										
**Major depression polygenic risk score**	0.00 [−0.01, 0.01] (0.8)	0.01 [0.00, 0.02] (0.2)	0.00 [−0.01, 0.01] (0.8)	**0.03** [0.02, 0.04] (1 × 10^−5^)	**−0.03** [−0.04, −0.02] (1 × 10^−5^)	**0.03** [0.01, 0.04] (4 × 10^−5^)	**−0.03** [−0.04, −0.02] (1 × 10^−5^)	**0.04** [0.02, 0.05] (3 × 10^−8^)	**0.03** [0.02, 0.05] (7 × 10^−7^)	0.03 [0.02, 0.04] (6 × 10^−6^)
**Bipolar disorder polygenic risk score**	0.01 [−0.01, 0.02] (0.4)	**0.02** [0.01, 0.03] (0.004)	0.01 [0.00, 0.02] (0.2)	0.01 [0.00, 0.02] (0.2)	0.00 [−0.02, 0.01] (0.5)	0.01 [0.00, 0.02] (0.1)	0.00 [−0.01, 0.01] (0.9)	0.01 [−0.01, 0.02] (0.3)	**0.02** [0.00, 0.03] (0.02)	0.01 [0.00, 0.03] (0.07)
**Schizophrenia polygenic risk score**	0.01 [0.00, 0.03] (0.07)	**0.03** [0.01, 0.04] (8 × 10^−5^)	0.00 [−0.01, 0.01] (0.9)	**0.02** [0.01, 0.04] (0.001)	**−0.02** [−0.03, −0.01] (0.007)	**0.02** [0.01, 0.04] (0.0006)	**−0.02** [−0.03, −0.01] (0.003)	**0.04** [0.03, 0.05] (3 × 10^−9^)	**0.05** [0.03, 0.06] (1 × 10^−11^)	**0.06** [0.04, 0.07] (1 × 10^−17^)

Covariate-corrected linear regression effect sizes (standardized β coefficients) and *p*-values for association between each sleep measure and each psychiatric diagnosis, across the 86,513 self-reported white participants with accelerometry. Bold denotes significant associations at 5% FDR; square brackets denote 95% confidence intervals; rounded brackets denote *p*-values.

FDR, false discovery rate; WASO, wake after sleep onset.

First, sleep pattern differences were ubiquitous across diagnoses. Having any psychiatric diagnosis was significantly associated with differences in every sleep measure except for total sleep duration, and each individual psychiatric diagnosis was associated with a median of 8.5 of the 10 sleep measures, though effect sizes were generally small. For instance, the largest magnitude effect size across the 4 disorders was β = −0.11 (95% confidence interval −0.13 to −0.10, *p* = 3 × 10^−56^, FDR = 6 × 10^−55^) for the association between lifetime inpatient major depressive disorder diagnosis and sleep efficiency.

Second, almost all significant associations with accelerometer-derived sleep measures and 18 significant associations with self-reported sleep properties had the same effect size directions: toward later bedtime and wake-up time; shorter duration of longest sleep bout; lower sleep efficiency; higher WASO and number of awakenings; more naps; and more variable bedtime and sleep duration. The one exception was sleep duration, which was significantly shorter among participants with lifetime major depressive disorder diagnoses (β = −0.02, 95% confidence interval −0.04, −0.01, *p* = 0.003, FDR = 0.003) but significantly longer among participants with lifetime schizophrenia spectrum disorder diagnoses (β = 0.02, 95% confidence interval 0.01 to 0.04, *p* = 0.0008, FDR = 0.001).

Third, despite this relative homogeneity, certain sleep properties were associated to a greater extent than others with lifetime psychopathology. In particular, across diagnoses, measures of sleep quality were more strongly associated than mere sleep duration. In particular, WASO, sleep efficiency, and number of awakenings were each associated with every tested disorder. In contrast, sleep duration was only significantly associated with major depressive disorder and schizophrenia spectrum disorders (see previous paragraph), and its effect size for major depressive disorder was several times smaller than for the other 9 sleep measures.

### Association of accelerometer-derived sleep measures with polygenic risk scores

To ascertain genetic influences on sleep patterns, we next associated each of the 10 accelerometer-derived sleep measures with polygenic risk scores for major depression, bipolar disorder, and schizophrenia (**Table 4**). Given the imperfect nature of polygenic risk scores, the effect sizes for these associations were generally smaller than for the psychiatric diagnoses; but since every individual in the cohort has a polygenic risk score (even though most lack psychiatric diagnoses), many were nonetheless significant, particularly for major depression and schizophrenia. As with the psychiatric diagnoses, all significant associations were in the direction of later wake-up time; shorter duration of longest sleep bout; lower sleep efficiency; higher WASO and number of awakenings; more naps; and more variable bedtime and sleep duration. Bedtime and sleep duration were not associated with any of the 3 polygenic risk scores.

### Replication across ancestries and sexes

Finally, we confirmed replicability of the associations between sleep measures and psychiatric diagnoses across ancestries and sexes (**S4 Table**). Due to the relatively low numbers of self-reported non-white participants in the sample, we restricted ourselves to replicating the “Any psychiatric diagnosis” row from **Table 4**. We found that, of the 10 accelerometer-derived sleep measures with significant associations among self-reported white participants, 3 measures replicated in self-reported non-white participants; 6 of the 7 associations that failed to replicate (all except for sleep duration) nonetheless had the same effect directions as in self-reported white participants. Replication among self-reported white males and females was better powered: all 10 significant associations with accelerometer-derived sleep measures replicated in both males and females, with comparable effect sizes to the non-sex stratified analysis.

### Comparison with self-reported sleep properties

As a secondary analysis, we considered 6 self-reported sleep properties—sleep duration, ease of morning awakening, chronotype, daytime napping, insomnia, and daytime dozing (**S1 Table**)—ascertained at baseline assessment between 2006 and 2010, approximately a half decade earlier than the accelerometry. We found that self-reported sleep properties were broadly concordant with their closest self-reported equivalents (**S2 Table**)—though not completely so, as expected given the known discordance between subjective and objective sleep measures, differences in the definitions of the 2 types of measures, and the half-decade time lag between the two. Among notable associations, self-reported sleep duration was most strongly associated with accelerometry-derived sleep duration (β = 0.40, 95% confidence interval 0.39 to 0.42, *p* = 0, FDR = 0); self-reported ease of morning awakening with accelerometry-derived wake-up time (β = −0.53, 95% confidence interval −0.54 to −0.51, *p* = 0, FDR = 0); self-reported chronotype (higher values indicate one is more of an “evening person” than a “morning person”) with accelerometry-derived bedtime (β = 0.69, 95% confidence interval 0.67 to 0.70, *p* = 0, FDR = 0) and wake-up time (β = 0.73, 95% confidence interval 0.72 to 0.75, *p* = 0, FDR = 0); and self-reported daytime napping with accelerometry-derived number of naps (β = 0.38, 95% confidence interval 0.37 to 0.40, *p* = 0, FDR = 0).

Next, we performed the same battery of associations with psychiatric diagnoses and polygenic risk scores on these self-reported sleep properties (**S3 Table**) as for the accelerometry-derived sleep measures. Despite much stronger statistical significance due to the increased sample size, effect sizes were not substantially larger than for the primary analysis (**Table 4**). For instance, the largest magnitude effect size across the 4 disorders was β = −0.12 (95% confidence interval −0.12 to −0.11, *p* = 6 × 10^−258^, FDR = 6 × 10^−257^) for the association between lifetime inpatient major depressive disorder diagnosis and ease of morning awakening, essentially identical to the largest effect size for the accelerometry-derived measures (β = −0.11 for the association between major depressive disorder and sleep efficiency, as mentioned above).

## Discussion

In this work, we analyzed the structure of sleep and its association with lifetime psychopathology across nearly 90,000 individuals. In a departure from previous studies analyzing only a single sleep property or a single disorder, we take an “all-by-all” approach, associating 10 accelerometer-derived sleep measures with 4 inpatient psychiatric diagnoses and 3 psychiatric polygenic risk scores. On the whole, accelerometer-derived sleep measures were concordant with self-reported sleep properties, and both were richly associated with psychiatric diagnoses and polygenic risk scores, and these associations replicated across ancestries and sexes. To our knowledge, this is the first large-scale transdiagnostic study of objectively measured sleep and mental health.

The same sleep pattern differences tended to recur across disorders: each diagnosis was associated with a median of 8.5 of the 10 sleep measures, almost always in the direction of worse sleep quality. However, effect sizes were generally quite small. Note that these numbers are with respect to lifetime diagnoses; the extent of sleep disruption would presumably be greater during an active episode of depression, mania, or psychosis [19].

Across diagnoses, metrics pertaining to sleep quality were more strongly associated than mere sleep duration. Strikingly, the accelerometry-defined duration of an individual’s longest sleep bout was much more strongly associated with most psychiatric diagnoses and polygenic risk scores than total sleep duration. Given the intimate relationship between sleep bout length and sleep quality [46,47], this suggests that sleep quality may be more disturbed than sleep length across psychopathologies. These findings undergird the importance of assessment of sleep quality in addition to sleep duration. However, we note that effects on sleep may vary greatly across disease subtypes (for instance, atypical versus nonatypical depression) or states (for instance, manic episode versus depressive episode versus euthymia), and these effects may be obscured when lumping together subtypes and states, as we do here.

Most prior studies of sleep and mental illness have focused on white individuals, and a key differentiating factor of our work is its replication across diverse ancestries, including those historically underrepresented in medical research [48]. In addition to this *trans*-ethnic replication, we also confirm that males and females display similar sleep alterations across lifetime psychopathologies. Even so, our results should be interpreted in the context of the UK Biobank’s well-characterized “healthy volunteer” selection bias [49] and its consequent underascertainment of individuals with psychiatric diagnoses [50].

This study has several key limitations. First, it relies on linked inpatient medical records, which may not capture all participants with clinically significant psychopathology, thus compounding the “healthy volunteer” bias mentioned in the previous paragraph. Second, the (often years-long) time lag between psychiatric diagnoses and accelerometry (**Table 2**) obscures whether participants were in an active manic, depressive, or psychotic episode at the time of their accelerometry. Third, the study’s cross-sectional design limits the ability to make inferences about causality. Fourth, accelerometer-based sleep measurement is not as precise as polysomnography, the gold standard in sleep research. The algorithm used for sleep/wake segmentation [32,33] was trained on accelerometry data annotated from head-mounted video and sleep diaries, rather than direct measures of sleep/wake, which could result in the misclassification of certain awake-in-bed periods (for instance, short awakenings or periods prior to sleep onset where the individual is motionless) as sleep. This may also account for the relatively high median sleep efficiency, low wake time after sleep onset, and long sleep bout durations seen in this study relative to polysomnography-based studies [51]. Also, accelerometry alone cannot accurately distinguish between rapid eye movement (REM) sleep and the various stages of non-REM sleep [52,53]. However, these limitations should be weighted against the population-scale, pan-diagnostic scope that accelerometry-based sleep measurement enables. Moreover, certain of our sleep metrics may indirectly capture aspects of sleep stage: for instance, high numbers of awakenings or low duration of longest sleep bout may indicate insufficient REM sleep [46,47,54].

A key clinical implication of this work is that sleep pattern differences are a transdiagnostic feature of psychopathology. Alterations in sleep parameters—particularly those impacting sleep quality and not merely duration—should be considered regardless of which psychiatric conditions a patient presents with. Future transdiagnostic studies of sleep and psychopathology should employ a longitudinal design to more precisely examine how sleep parameters vary across phases of mental illness.

In sum, we find that alterations in objectively measured sleep parameters are the norm among patients with lifetime psychiatric illness. Our findings provide a rich clinical portrait of the ways in which sleep can be disrupted across individuals with lifetime mental illness. This work showcases the capacity of accelerometry to provide detailed, objective sleep measurements at scale, even across cohorts of tens of thousands of individuals.

## Supporting information

S1 Checklist STROBE checklist. (DOC)

S1 Fig AUC curves summarizing the predictive accuracy of our polygenic risk scores at various GWAS *p*-value thresholds. The AUC, also known as the AUROC or C statistic, is the fraction of the time that the polygenic risk score would rank a randomly chosen case higher than a randomly chosen control. Polygenic risk scores were benchmarked against ICD codes from inpatient, primary care, or death records for the corresponding disorder (using the UK Biobank’s “Source of report of [ICD code]” fields; **Methods**): for major depressive disorder, F32 or F33; for bipolar disorder, F31; and for schizophrenia, F20. AUCs were computed across 451,993 self-reported white participants in the UK Biobank with genotype data. AUC, area under the curve; AUROC, area under the receiver operating characteristic curve; C statistic, concordance statistic; GWAS, genome-wide association study; ICD, International Classification of Diseases.
(EPS)

S2 Fig Correlations among the 10 accelerometer-derived sleep measures. Semipartial Kendall correlation coefficients (τ) among all pairs of accelerometer-derived sleep measures, hierarchically clustered with Euclidean distance and average linkage.
(EPS)

S1 Table The 6 self-reported sleep properties. (DOCX)

S2 Table Concordance of accelerometer-derived sleep measures (columns) with self-reported sleep properties (rows). Covariate-corrected linear regression effect sizes (β coefficients) and *p*-values for association between each accelerometer-derived sleep measure and each self-reported sleep property and each psychiatric diagnosis, across the 77,232 self-reported white participants with both types of sleep properties. Bold denotes significant associations at 5% FDR; square brackets denote 95% confidence intervals; rounded brackets denote *p*-values. WASO, wake after sleep onset.
(DOCX)

S3 Table Association of self-reported sleep properties with psychiatric diagnoses and polygenic risk scores. Covariate-corrected linear regression effect sizes (standardized β coefficients) and *p*-values for association between each self-reported sleep property and each psychiatric diagnosis, across the 400,771 self-reported white participants with self-reported sleep properties. Bold denotes significant associations at 5% FDR; square brackets denote 95% confidence intervals; rounded brackets denote *p*-values. FDR, false discovery rate.
(DOCX)

S4 Table Replication of associations between accelerometer-derived sleep measures and lifetime psychopathology. Replication of the “Any psychiatric diagnosis” row of Table 4, both in non-white participants (67 participants of 2,692 have at least one of the 4 inpatient psychiatric diagnoses) and stratified by sex (1,445 females out of 48,562 and 757 males out of 37,951 have at least one of the 4). Covariate-corrected linear regression effect sizes (β coefficients) and *p*-values are shown for each sleep measure. Bold denotes significant associations at 5% FDR; square brackets denote 95% confidence intervals; rounded brackets denote *p*-values. FDR, false discovery rate; WASO, wake after sleep onset.
(DOCX)

## Abbreviations

FDR: false discovery rate
HDCZA: Heuristic algorithm looking at Distribution of Change in Z-Angle
ICD: International Classification of Diseases
LD: linkage disequilibrium
REM: rapid eye movement
WASO: wake after sleep onset
