# Supplementary material for: Association of accelerometer-derived sleep measures with lifetime psychiatric diagnoses: A cross-sectional study of 89,205 participants from the UK Biobank
Source: PLoS Med. 2021 Oct 12;18(10):e1003782. doi: 10.1371/journal.pmed.1003782 (PMC8509859; doi:10.1371/journal.pmed.1003782)
Supplement: S1 Table — (DOCX) [file pmed.1003782.s004.docx]

| **Phenotype** | **Field ID** | **Type** | **Question phrasing (for self-report phenotypes)** |
| --- | --- | --- | --- |
| Sleep duration | 1160 | Ordinal (1 to 23 hours, in 1-hour increments) | About how many hours’ sleep do you get in every 24 hours? (please include naps) |
| Getting up in morning  (“Ease of morning awakening”) | 1170 | Ordinal (1 = Not at all easy; 2 = Not very easy; 3 = Fairly easy; 4 = Very easy) | On an average day, how easy do you find getting up in the morning? |
| Morning/evening person (chronotype) | 1180 | Ordinal (1 = Definitely a morning person; 2 = More a morning than evening person; 3 = More an evening than a morning person; 4 = Definitely an evening person) | Do you consider yourself to be: definitely a morning person, more a morning than evening person, more an evening than a morning person, or definitely an evening person? |
| Nap during day | 1190 | Ordinal (1 = Never/rarely; 2 = Sometimes; 3 = Usually) | Do you have a nap during the day? |
| Sleeplessness/insomnia | 1200 |  | Do you have trouble falling asleep at night or do you wake up in the middle of the night? |
| Daytime dozing/sleeping (narcolepsy) | 1220 | Ordinal (0 = Never/rarely; 1 = Sometimes; 2 = Often; 3 = All of the time) | How likely are you to doze off or fall asleep during the daytime when you don’t mean to? (e.g. when working, reading or driving) |

**S1 Table: The 6 self-reported sleep properties.**
