# Supplementary material for: Association of accelerometer-derived sleep measures with lifetime psychiatric diagnoses: A cross-sectional study of 89,205 participants from the UK Biobank
Source: PLoS Med. 2021 Oct 12;18(10):e1003782. doi: 10.1371/journal.pmed.1003782 (PMC8509859; doi:10.1371/journal.pmed.1003782)
Supplement: S2 Table — Covariate-corrected linear regression effect sizes (β coefficients) and p-values for association between each accelerometer-derived sleep measure and each self-reported sleep property and each psychiatric diagnosis, across the 77,232 self-reported white participants with both types of sleep properties. Bold denotes significant associations at 5% FDR; square brackets denote 95% confidence intervals; rounded brackets denote p-values. WASO, wake after sleep onset. (DOCX) [file pmed.1003782.s005.docx]

|  | **Bedtime** | **Wake-up time** | **Sleep duration** | **Wake after sleep onset** | **Sleep efficiency** | **# awakenings** | **Longest sleep bout** | **# naps** | **Bedtime variability** | **Sleep duration variability** |
| --- | --- | --- | --- | --- | --- | --- | --- | --- | --- | --- |
| **Sleep duration** | **-0.20**  [-0.21, -0.18]  (6 × 10^-163^) | **0.23**  [0.22, 0.24]  (3 × 10^-223^) | **0.40**  [0.39, 0.42]  (0) | 0.01  [-0.01, 0.02]  (0.4) | **0.03**  [0.02, 0.04]  (4 × 10^-5^) | **0.03**  [0.02, 0.04]  (2 × 10^-5^) | **0.23**  [0.21, 0.24]  (1 × 10^-220^) | **-0.02**  [-0.03, -0.01]  (0.007) | **-0.05**  [-0.07, -0.04]  (4 × 10^-13^) | **-0.05**  [-0.06, -0.03]  (3 × 10^-11^) |
| **Ease of morning awakening** | **-0.40**  [-0.42, -0.39]  (0) | **-0.53**  [-0.54, -0.51]  (0) | **-0.05**  [-0.06, -0.04]  (2 × 10^-12^) | -0.01  [-0.03, -0.00]  (0.05) | **0.02**  [0.00, 0.03]  (0.01) | **-0.02**  [-0.04, -0.01]  (0.003) | 0.01  [-0.01, 0.02]  (0.3) | **-0.04**  [-0.05, -0.02]  (6 × 10^-8^) | **-0.04**  [-0.06, -0.03]  (6 × 10^-9^) | **-0.08**  [-0.09, -0.06]  (7 × 10^-28^) |
| **Chronotype** | **0.69**  [0.67, 0.70]  (0) | **0.73**  [0.72, 0.75]  (0) | **-0.03**  [-0.05, -0.02]  (3 × 10^-6^) | **-0.05**  [-0.07, -0.04]  (8 × 10^-14^) | **0.04**  [0.03, 0.06]  (1 × 10^-9^) | **-0.05**  [-0.06, -0.03]  (1 × 10^-10^) | -0.01  [-0.03, -0.00]  (0.05) | **0.04**  [0.02, 0.05]  (5 × 10^-7^) | **0.06**  [0.04, 0.07]  (1 × 10^-14^) | **0.06**  [0.05, 0.08]  (5 × 10^-18^) |
| **Daytime napping** | **0.06**  [0.04, 0.07]  (5 × 10^-15^) | **-0.04**  [-0.05, -0.03]  (1 × 10^-8^) | **-0.14**  [-0.15, -0.13]  (3 × 10^-83^) | **0.16**  [0.14, 0.17]  (6 × 10^-105^) | **-0.18**  [-0.19, -0.17]  (8 × 10^-137^) | **0.14**  [0.13, 0.16]  (1 × 10^-86^) | **-0.20**  [-0.22, -0.19]  (9 × 10^-173^) | **0.38**  [0.37, 0.40]  (0) | **0.12**  [0.11, 0.14]  (1 × 10^-64^) | **0.11**  [0.09, 0.12]  (1 × 10^-49^) |
| **Insomnia** | **-0.06**  [-0.07, -0.05]  (8 × 10^-17^) | **0.02**  [0.01, 0.04]  (0.004) | **0.03**  [0.02, 0.04]  (3 × 10^-5^) | **0.16**  [0.15, 0.18]  (3 × 10^-115^) | **-0.18**  [-0.19, -0.16]  (1 × 10^-132^) | **0.21**  [0.20, 0.23]  (2 × 10^-194^) | **-0.17**  [-0.19, -0.16]  (8 × 10^-129^) | **0.07**  [0.05, 0.08]  (1 × 10^-21^) | **0.06**  [0.05, 0.08]  (1 × 10^-18^) | **0.07**  [0.06, 0.08]  (8 × 10^-23^) |
| **Daytime dozing** | **0.12**  [0.11, 0.14]  (9 × 10^-67^) | **-0.07**  [-0.08, -0.05]  (3 × 10^-21^) | **-0.21**  [-0.22, -0.19]  (6 × 10^-183^) | **0.08**  [0.07, 0.09]  (2 × 10^-28^) | **-0.11**  [-0.12, -0.10]  (4 × 10^-53^) | **0.06**  [0.05, 0.08]  (3 × 10^-18^) | **-0.19**  [-0.20, -0.17]  (3 × 10^-147^) | **0.17**  [0.15, 0.18]  (2 × 10^-118^) | **0.09**  [0.08, 0.10]  (2 × 10^-35^) | **0.06**  [0.05, 0.08]  (2 × 10^-18^) |

**S2 Table: Concordance of accelerometer-derived sleep measures (columns) with self-reported sleep properties (rows).** Covariate-corrected linear regression effect sizes (standardized β coefficients) and p-values for association between each accelerometer-derived sleep measure and each self-reported sleep property and each psychiatric diagnosis, across the 77,232 self-reported white participants with both types of sleep properties. Bold denotes significant associations at 5% FDR; square brackets denote 95% confidence intervals; rounded brackets denote p-values.
