# Supplementary material for: Association of accelerometer-derived sleep measures with lifetime psychiatric diagnoses: A cross-sectional study of 89,205 participants from the UK Biobank
Source: PLoS Med. 2021 Oct 12;18(10):e1003782. doi: 10.1371/journal.pmed.1003782 (PMC8509859; doi:10.1371/journal.pmed.1003782)
Supplement: S3 Table — Covariate-corrected linear regression effect sizes (standardized β coefficients) and p-values for association between each self-reported sleep property and each psychiatric diagnosis, across the 400,771 self-reported white participants with self-reported sleep properties. Bold denotes significant associations at 5% FDR; square brackets denote 95% confidence intervals; rounded brackets denote p-values. FDR, false discovery rate. (DOCX) [file pmed.1003782.s006.docx]

|  | **Sleep duration** | **Ease of morning awakening** | **Chronotype** | **Daytime napping** | **Insomnia** | **Daytime dozing** |
| --- | --- | --- | --- | --- | --- | --- |
| **Psychiatric diagnoses** | | | | | | |
| **Any psychiatric diagnosis**  **N = 5424** | **0.04**  [0.03, 0.05]  (2 × 10^-33^) | **-0.14**  [-0.14, -0.13]  (0) | **0.05**  [0.05, 0.06]  (2 × 10^-51^) | **0.11**  [0.10, 0.11]  (4 × 10^-209^) | **0.09**  [0.08, 0.09]  (3 × 10^-141^) | **0.09**  [0.08, 0.09]  (8 × 10^-138^) |
| **Major depressive disorder (F32-F33)**  **N = 3665** | **0.02**  [0.02, 0.03]  (7 × 10^-11^) | **-0.12**  [-0.12, -0.11]  (6 × 10^-258^) | **0.04**  [0.03, 0.05]  (1 × 10^-34^) | **0.09**  [0.08, 0.09]  (5 × 10^-145^) | **0.08**  [0.07, 0.09]  (1 × 10^-122^) | **0.07**  [0.06, 0.08]  (5 × 10^-98^) |
| **Anxiety (F40-F41)**  **N = 1358** | **0.01**  [0.00, 0.01]  (0.04) | **-0.05**  [-0.06, -0.05]  (8 × 10^-62^) | **0.02**  [0.02, 0.03]  (4 × 10^-12^) | **0.04**  [0.03, 0.04]  (9 × 10^-28^) | **0.04**  [0.03, 0.05]  (3 × 10^-32^) | **0.04**  [0.03, 0.04]  (1 × 10^-27^) |
| **Bipolar/mania**  **(F30-F31)**  **N = 540** | **0.03**  [0.02, 0.04]  (2 × 10^-22^) | **-0.03**  [-0.04, -0.02]  (5 × 10^-20^) | **0.01**  [0.01, 0.02]  (2 × 10^-5^) | **0.04**  [0.04, 0.05]  (2 × 10^-40^) | 0.01  [0.00, 0.01]  (0.09) | **0.03**  [0.02, 0.03]  (4 × 10^-16^) |
| **Schizophrenia**  **spectrum (F20-F29)**  **N = 524** | **0.05**  [0.05, 0.06]  (6 × 10^-59^) | **-0.04**  [-0.04, -0.03]  (4 × 10^-34^) | **0.02**  [0.02, 0.03]  (2 × 10^-11^) | **0.04**  [0.03, 0.04]  (3 × 10^-34^) | **0.01**  [0.00, 0.02]  (0.0005) | **0.02**  [0.02, 0.03]  (2 × 10^-11^) |
| **Psychiatric polygenic risk scores** | | | | | | |
| **Major depression**  **polygenic risk score** | **-0.01**  [-0.02, -0.01]  (1 × 10^-5^) | **-0.04**  [-0.05, -0.04]  (5 × 10^-39^) | **0.01**  [0.01, 0.02]  (0.0002) | **0.03**  [0.02, 0.04]  (4 × 10^-22^) | **0.06**  [0.05, 0.07]  (6 × 10^-80^) | **0.02**  [0.02, 0.03]  (1 × 10^-11^) |
| **Bipolar disorder polygenic risk score** | **0.02**  [0.01, 0.02]  (4 × 10^-7^) | **-0.02**  [-0.02, -0.01]  (3 × 10^-8^) | **0.01**  [0.00, 0.01]  (0.03) | **0.02**  [0.02, 0.03]  (5 × 10^-12^) | 0.01  [0.00, 0.01]  (0.1) | **0.01**  [0.01, 0.02]  (0.0002) |
| **Schizophrenia polygenic risk score** | **0.05**  [0.04, 0.05]  (1 × 10^-46^) | **-0.07**  [-0.07, -0.06]  (1 × 10^-99^) | **0.03**  [0.03, 0.04]  (5 × 10^-26^) | **0.05**  [0.04, 0.06]  (8 × 10^-57^) | 0.00  [0.00, 0.01]  (0.2) | **0.02**  [0.02, 0.03]  (7 × 10^-13^) |

**S3 Table: Association of self-reported sleep properties with psychiatric diagnoses and polygenic risk scores.** Covariate-corrected linear regression effect sizes (standardized β coefficients) and p-values for association between each self-reported sleep property and each psychiatric diagnosis, across the 400,771 self-reported white participants with self-reported sleep properties. Bold denotes significant associations at 5% FDR; square brackets denote 95% confidence intervals; rounded brackets denote p-values.
