# Supplementary material for: Association of accelerometer-derived sleep measures with lifetime psychiatric diagnoses: A cross-sectional study of 89,205 participants from the UK Biobank
Source: PLoS Med. 2021 Oct 12;18(10):e1003782. doi: 10.1371/journal.pmed.1003782 (PMC8509859; doi:10.1371/journal.pmed.1003782)
Supplement: S4 Table — Replication of the “Any psychiatric diagnosis” row of Table 4, both in non-white participants (67 participants of 2,692 have at least one of the 4 inpatient psychiatric diagnoses) and stratified by sex (1,445 females out of 48,562 and 757 males out of 37,951 have at least one of the 4). Covariate-corrected linear regression effect sizes (β coefficients) and p-values are shown for each sleep measure. Bold denotes significant associations at 5% FDR; square brackets denote 95% confidence intervals; rounded brackets denote p-values. FDR, false discovery rate; WASO, wake after sleep onset. (DOCX) [file pmed.1003782.s007.docx]

|  | **Bedtime** | **Wake-up time** | **Sleep duration** | **Wake after sleep onset** | **Sleep efficiency** | **# awakenings** | **Longest sleep bout** | **# naps** | **Bedtime variability** | **Sleep duration variability** |
| --- | --- | --- | --- | --- | --- | --- | --- | --- | --- | --- |
| **Self-reported non-white** | 0.01  [-0.07, 0.09]  (0.7) | 0.06  [-0.02, 0.14]  (0.2) | 0.03  [-0.05, 0.11]  (0.5) | **0.20**  [0.12, 0.28]  (6 × 10^-7^) | **-0.20**  [-0.28, -0.13]  (5 × 10^-7^) | **0.13**  [0.05, 0.21] (0.001) | -0.06  [-0.14, 0.02]  (0.1) | 0.08  [0.00, 0.16] (0.05) | 0.03  [-0.05, 0.11]  (0.5) | 0.08  [0.00, 0.16]  (0.06) |
| **Self-reported white, female**  (1445 cases out of 48,562) | **0.09**  [0.08, 0.11]  (3 × 10^-22^) | **0.13**  [0.11 ,0.15]  (3 × 10^-39^) | **-0.02**  [-0.04, 0.00]  (0.04) | **0.12**  [0.10, 0.14]  (3 × 10^-36^) | **-0.15**  [-0.17, -0.13]  (1 × 10^-53^) | **0.12**  [0.11, 0.14]  (5 × 10^-37^) | **-0.12**  [-0.14, -0.10]  (2 × 10^-33^) | **0.12**  [0.10, 0.14]  (4 × 10^-34^) | **0.08**  [0.06, 0.10]  (2 × 10^-15^) | **0.10**  [0.09, 0.12]  (9 × 10^-27^) |
| **Self-reported, white, male**  (757 cases out of 37,951) | **0.05**  [0.03, 0.07]  (5 × 10^-6^) | **0.05**  [0.03, 0.08]  (4 × 10^-7^) | **-0.04**  [-0.06, -0.02]  (0.0003) | **0.11**  [0.09, 0.13]  (4 × 10^-24^) | **-0.13**  [-0.15, -0.11]  (1 × 10^-32^) | **0.10**  [0.08, 0.12]  (2 × 10^-20^) | **-0.10**  [-0.12, -0.08]  (8 × 10^-20^) | **0.12**  [0.10, 0.14]  (3 × 10^-27^) | **0.09**  [0.07, 0.11]  (1 × 10^-15^) | **0.10**  [0.08, 0.13]  (2 × 10^-22^) |

**S4 Table: Replication of associations between accelerometer-derived sleep measures and lifetime psychopathology.** Replication of the "Any psychiatric diagnosis" row of Table 4, both in non-white participants (67 participants of 2692 have at least one of the four inpatient psychiatric diagnoses) and stratified by sex (1445 females out of 48,562 and 757 males out of 37,951 have at least one of the four). Covariate-corrected linear regression effect sizes (standardized β coefficients) and p-values are shown for each sleep measure. Bold denotes significant associations at 5% FDR; square brackets denote 95% confidence intervals; rounded brackets denote p-values.
